# Degradation of catecholate, hydroxamate, and carboxylate model siderophores by extracellular enzymes

**DOI:** 10.1371/journal.pone.0330432

**Published:** 2025-08-19

**Authors:** Chinazam Emmanuel Chukwuma, Owen William Duckworth, Oliver Baars

**Affiliations:** 1 Department of Chemistry, North Carolina State University, Raleigh, North Carolina, United States of America; 2 Department of Crop and Soil Sciences, North Carolina State University, Raleigh, North Carolina, United States of America; 3 Department of Entomology and Plant Pathology, North Carolina State University, Raleigh, North Carolina, United States of America; Graphic Era Institute of Technology: Graphic Era Deemed to be University, INDIA

## Abstract

Siderophores are low-molecular weight biomolecules with a high affinity for ferric iron (Fe^III^) that can impact plant and microbial growth. Although their formation and biology have been investigated in detail, little is known about the environmental fate of siderophores, including their potential reactions with common degradative enzymes, which may influence or hinder the ability to promote the uptake of Fe for plants and microbes. In this study, we examined the ability of the model extracellular enzymes phenol oxidase, protease, and horseradish peroxidase to degrade apo siderophores and Fe^III^ siderophore complexes. The siderophores were selected to represent the natural diversity of siderophore structures: the bacterial triscatecholamide siderophore protochelin; the bacterial trishydroxamate siderophore desferrioxamine B (DFOB); and the synthetic carboxylate phytosiderophore analog proline-2′-deoxymugineic acid (PDMA). In general, apo siderophores were more susceptible to degradation, with some protection of the siderophore provided by Fe^III^ complexation. Phenol oxidase reacted rapidly with protochelin, leading to 90% degradation of protochelin after 24 hours of reaction, which could be modeled by Michaelis-Menten kinetics. Peroxidases in the presence of H_2_O_2_ were also effective in the degradation of protochelin (80%) and, to a lesser extent, reacted with DFOB, leading to ~5% degradation. Control experiments showed that protochelin oxidation is caused primarily by H_2_O_2_ alone, even in the absence of the peroxidase enzyme. When bound to Fe^III^, the degradation of protochelin by phenol oxidase and DFOB degradation by peroxidase was reduced by ~50% and ~3%, respectively. No significant reaction was detected between PDMA and any of the three enzymes, supporting its proposed use for plant Fe fertilization.

## Introduction

Iron (Fe) is an essential nutrient for plant growth and crop production. Competition for Fe impacts microbial activity and host-pathogen interactions [1,2]. At circumneutral pH and in oxygenated environments, Fe bioavailability is restricted by the formation of low solubility Fe^III^ oxide and hydroxide minerals. Bacteria [3,4], fungi [5], and graminaceous plants [2,6–8] exude siderophores, a structurally diverse class of small molecules, to solubilize, complex, and promote the uptake of Fe from otherwise biologically unavailable sources. Iron acquisition with siderophores is typically conceptualized to occur via the following steps: (1) siderophore biosynthesis and secretion [2,9]; (2) diffusion of the free siderophore in the extracellular medium [10–12]; (3) complexation of Fe^III^ from environmental sources [13–15]; (4) diffusion of the Fe^III^-siderophore complex back to the cell surface [16,17]; and (5) Fe uptake from the Fe^III^-siderophore complex via processes such as ferric siderophore transporters or reductive Fe uptake [18,19].

Through the transport and solubilization processes, there may be numerous sinks for siderophores that disrupt Fe uptake [20–22]. Among the possible siderophore sinks are reactions with environmentally common extracellular enzymes [6,13,23]. Extracellular enzymes may have high activities at circumneutral pH, as plants and microbes secrete them to promote the degradation of organic matter, lignin, and phenolic compounds [24], and the cycling of nutrients [25,26].

At present, we have a poor understanding of how common degradative extracellular enzymes may react with siderophores. Previously reported examples of enzymatic siderophore degradation have focused on intracellular processes that allow microbes to utilize siderophores as a carbon substrate. The reported degradation reactions involved the hydrolysis of the amide bonds in the siderophores desferrioxamine B [27,28] and ferrichrome A [29–31]. In addition to siderophore degradation for carbon acquisition, some bacteria may utilize enzymes to release Fe^III^ from high affinity triscatecholate siderophores. For example, *Escherichia coli* expresses ferric enterobactin esterase to hydrolyze the enterobactin trilactone scaffold, promoting bioavailability of the bound Fe [12,32,33]. More recently, a reduction of hydroxamate groups in trishydroxamate siderophores was reported in cultures of the root wheat symbiont *Pyrenophora biseptata* [34].

In this study, we specifically investigated whether selected model extracellular enzymes were able to react with and possibly degrade apo-siderophores and Fe^III^-siderophore complexes. The three chosen model enzymes included phenol oxidase (a tyrosinase) from the cultivated mushroom *Agaricus bisporus*, peroxidase from horseradish, and proteases from the bacterium *Streptomyces griseus*, all of which are known to be involved in organic matter decomposition and nutrient cycling [15,24,25,27,35–38]. It is worth noting that there are three phenol oxidase subtypes: catechol oxidases (catalyze the oxidation of o-diphenols), aurone synthases (oxidize chalcones to aurones, yellow pigment), and tyrosinases (hydroxylate monophenols and oxidize o-diphenols), the last of which we used in this study [39]. To represent the wide variety of siderophore structures and chemistries, we used three model siderophores: protochelin, a bacterial triscatecholamide siderophore; desferrioxamine B (DFOB), a bacterial trishydroxamate siderophore; and proline-2′-deoxymugineic acid (PDMA), a synthetic carboxylate phytosiderophore analog, developed as a novel effective iron fertilizer [40,41]. To reduce the cost of synthesis and increase the stability of PDMA over its natural counterpart, deoxymugineic acid, the azetidine-2-carboxylic acid group in DMA has been substituted with L-proline. Each of the three siderophores was reacted as the apo-siderophore and as the Fe^III^ complex with each of the three enzymes. The degradation was monitored over time by UV-visible spectrophotometry and liquid chromatography-mass spectrometry (LC-MS). The observed siderophore degradation depended not only on the siderophore structure but also on the binding state: the ferric complexes were less reactive than the apo siderophores. Reaction kinetics for the protochelin degradation reactions were rapid and suggested the potential for catechol siderophore degradation via extracellular enzymes in microbiomes; however, further studies are required to elucidate the impact of side reactions in different ecosystem matrices.

## Materials and methods

### Siderophores

**Fig 1:**
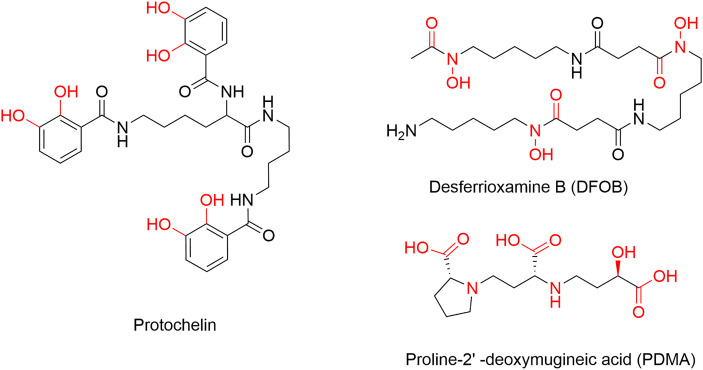
Structure of the three siderophores in this study. The moieties involved in Fe^III^ complexation at pH ~ 7 are indicated in red.

Protochelin was synthetically prepared by the Small Molecule Synthesis Facility (SMSF) at Duke University, as previously reported [21]. PDMA was provided as PDMA∙2HCl by Aichi Steel Corporation, Japan [40]. DFOB was purchased as deferoxamine mesylate salt from Sigma-Aldrich and used as received (Fig 1). Stock solutions of 10 mM were prepared in 10 mL of methanol for protochelin or 10 mL of deionized water (MQ) for DFOB and PDMA. Working solutions were prepared by diluting the stock solution to 100 µM with MQ water. To prepare the Fe^III^-siderophore complex, 100 µM FeCl_3_ was added to 100 µM of siderophore in MQ water. The mixture was left to stand for 1 hour in the dark with occasional shaking and then centrifuged at ca. 22000 × *g* for 3 minutes. Formation of Fe^III^-siderophore complexes was confirmed by spectrophotometric and liquid chromatography-mass spectrometry (LC-MS) analyses. Siderophore stocks were immediately used or stored for up to two weeks at −20 ºC.

### Enzymes and enzyme activity

The following three enzymes were used: (1) phenol oxidase isolated from the mushroom *Agaricus bisporus* (MilliporeSigma T3824, lyophilized powder; ≥ 1000 units/mg solid); (2) peroxidase from horseradish (Type II, MilliporeSigma P8250, 150–250 units/mg solid); and (3) protease from *Streptomyces griseus* (MilliporeSigma P5147, ≥ 3.5 units/mg). Protease from *Streptomyces griseus* consists of at least three proteolytic enzymes, including an extracellular serine protease. Assays to confirm enzyme activities were conducted using established protocols (see SI for details). Briefly, phenol oxidase activity was determined using a method adapted from [42] with L-tyrosine and oxygen as substrates, with a measured activity of 2420 units/mg solid. For peroxidase, a method adapted from [43] was used with pyrogallol and hydrogen peroxide as substrates. The measured activity was 55 units/mg after a 10-fold dilution of the enzyme stock solution. The protease activity was determined according to [44] and [45] with casein as substrate. The activity was found to be 7.5 units/mg solid powder.

### Siderophore reactions with enzymes

Each of the three siderophores (Fig 1) was reacted with each of the three enzymes, both as the apo-siderophore and Fe^III^-siderophore complex at pH = 7. For phenol oxidase and protease reactions, solutions were buffered with 1 mL of phosphate buffer (25 mM, pH = 7.0). A volume of 0.3 mL of phenol oxidase (454 units/mL) or protease (7.5 units/mL) was added. The reaction was started by the addition of 0.3 mL of a 100 µM stock solution (19 µM) of apo-siderophore or Fe^III^-siderophore complex. For peroxidase reactions, solutions were buffered with 0.5 mL of phosphate buffer (30 mM, pH = 6.8). A volume of 0.32 mL of the peroxidase enzyme solution (55 units/mL) was added. The reaction was started by adding 0.3 mL of a 100µM stock solution (23 µM) of apo-siderophore or the Fe^III^-siderophore complex and 0.16 mL of hydrogen peroxide (H_2_O_2_, 0.50%). Two further additions of 0.16 mL H_2_O_2_ were made at 1-hour intervals, which decreased the added siderophore concentrations to 19 µM. Controls consisted of: (1) no enzyme (addition of buffer solution instead of enzyme solution); (2) no siderophore; and (3) heat-inactivated enzyme reactions (where the enzyme was inactivated by heating to 95˚C for 20 minutes). In the case of peroxidase experiments, a fourth control consisted of the reaction mixture without adding H_2_O_2_, and water was added to maintain sample volume consistent with the concentrations of the enzyme and individual components.

Experiments were conducted in an incubator maintained at 20 ºC. Preliminary experiments monitored degradation by observation of absorption spectral changes. For peroxidase and H_2_O_2_, quantitative protochelin degradation occurred immediately with no further changes afterwards (S4 Fig). In contrast, phenol oxidase exhibited a slower reaction that seemed to follow Michaelis-Menten kinetics. Based on these results, reaction timepoints of immediate sampling, 0.5 h, 1 h, 2 h, and 24 h, were chosen to capture the rapid initial reaction, track slower degradation, detect degradation products accumulation, and observe potential secondary reactions or structural changes. A volume of 0.16 mL was sampled and analyzed with an ultraviolet-visible (UV-vis) spectrophotometer (190–700 nm, ThermoFisher Evolution 201). The reactions were quenched by the addition of 9 parts acetonitrile to 1 part of the enzyme reaction mixture to precipitate the enzymes, and samples were centrifuged (ca. 22000 × *g* for 10 min) and diluted 1:1 with MQ water for liquid chromatography-mass spectrometry (LC-MS) analysis. All experiments were conducted in triplicate.

### Enzyme kinetics and substrate specificity

The reaction between protochelin and phenol oxidase appeared to follow Michaelis-Menten kinetics. Experiments were conducted to determine the maximum reaction rate (V_max_) and Michaelis-Menten constant (K_m_). The parameters V_max_ and K_m_ were calculated by measuring initial reaction rates (v_0_) as a function of protochelin substrate concentrations (0.01 mM; 0.02 mM; 0.05 mM; 0.1 mM; 0.2 mM; 0.5 mM; 1 mM). The initial rate (v_0_) was defined as the slope of the initial linear portion of the curve obtained following the product formation as a function of time (0, 0.5, and 2 h). All measurements were done in triplicate.

### Analysis of siderophore reactions with liquid chromatography-mass spectrometry (LC-MS)

Samples were analyzed by LC-MS using a single quadrupole mass spectrometer (ISQ-EC, ThermoFisher) in combination with a diode array detector and charged aerosol detector (ThermoFisher). Separation was done with an Agilent Poroshell 120 EC-C18 column (4.6 × 100 mm, 2.7 µm) and a flow rate of 1.2 mL/min, and the column temperature was 30°C. A sample volume of 25 µL was injected and separated under a gradient of solvents A and B (A: water, 0.1% formic acid, 1% acetonitrile; B: acetonitrile, 0.1% formic acid, 2% water; gradient: 0–1.5 min 0% B, 1.5–8 min 0–100% B; 8–10 min 100% B; re-equilibration at 100% A for 4 min). Mass spectra were collected in single ion monitoring mode targeting the three added siderophores and potential degradation products (S1 Table). Siderophore degradation was followed by tracking peak areas in the enzymatic reactions and comparison to negative controls. To identify potential degradation products, select samples were analyzed with a high-resolution LC-MS/MS platform (Orbitrap Exploris 480, ThermoFisher). On the Orbitrap platform, the C18 column was a Restek Raptor C18 (2.1 × 100 mm) with a flow rate of 0.4 mL/min, and the column compartment was held at 45°C. Full-scan mass spectra (m/z = 85–1200) in positive ionization mode were collected with the resolution set to R = 60,000 (full width at half maximum at m/z = 400). Data-dependent MS/MS spectra were acquired for the top 5 most abundant ions in each cycle. Product ions were generated in HCD mode with 35 eV collision energy and an isolation window of 1.5 Da. The resolving power for MS/MS analysis was R = 15,000 (full width at half maximum at m/z = 400). Putative degradation products of the added siderophore standards were identified in high-resolution LC-MS data by searching for new LC-MS peaks in incubated samples and by mining of MS/MS spectra for characteristic siderophore groups (e.g., presence of 2,3-dihydroxybenzoid acid moieties in protochelin degradation products showing as neutral loss of *m/z* = 136.016 in MS/MS spectra [46]). Analysis of protochelin degradation products was also conducted by LC coupled to a UV-vis diode array detector by looking for new peaks with absorption maxima in the range of the absorption maxima of the 2,3-dihydroxybenzoid acid groups in protochelin (λ = 315 nm [47]).

## Results

### Decrease of dissolved siderophore concentrations by enzymes

**Fig 2:**
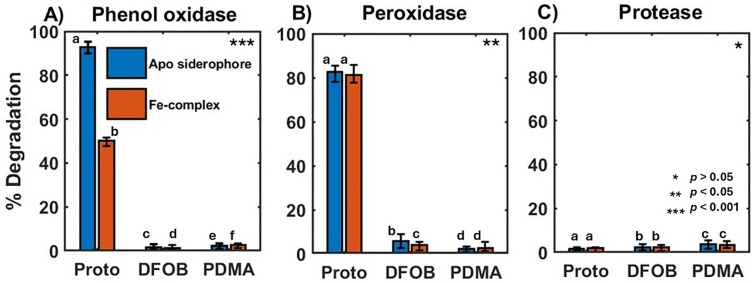
Percentage degradation of each of the three siderophores after reaction with (A) phenol oxidase, (B) peroxidase, and (C) protease enzymes. Blue and orange bars indicate the reaction with the apo siderophore and Fe^III^-siderophore complex, respectively. Statistical significance was determined using a one-way ANOVA with Tukey’s post hoc HSD test. Distinct letters indicate statistically significant differences between groups; asterisks (*) in the upper right corner of each plot denote the level of significance. Error bars show the range of measured degradation in three replicate experiments.

The phenol oxidase enzyme reacted with the triscatecholamide siderophore protochelin. The reaction with apo protochelin was nearly quantitative: ~ 90% of the initial protochelin concentration was not detected after a 24 h reaction period (Fig 2a and S1 Fig). Reaction with the Fe^III^-protochelin complex was slower or less complete: ~ 50% of the initial concentration had reacted after 24 h with phenol oxidase. Expectedly, DFOB and PDMA, which do not contain phenol or catechol groups (Fig 2a), did not react appreciably with the phenol oxidase. This trend showed statistically significant differences between apo siderophores and Fe^III^ siderophore complex reactions (p < 0.001) with phenol oxidases. In contrast, reactions with protease indicated no significant statistical difference (p ≥ 0.05) for apo siderophores and Fe^III^ siderophore complexes. Experimental controls of all siderophores with the phenol oxidase and protease enzymes showed no changes in absorbance spectra (data not shown).

The reaction of peroxidase and H_2_O_2_ with protochelin resulted in ~80% degradation of both apo-protochelin and Fe^III^-protochelin after a 24 h reaction period based on LC-MS quantification (Fig 2b). For the Fe^III^-protochelin complex, the characteristic blue color of the Fe-complex turned clear after a 24 h reaction alongside the disappearance of the UV-vis spectral signature of the protochelin Fe-complex (λ = 560 nm [47,48]). However, controls with heat-inactivated peroxidase and H_2_O_2_ used, or with H_2_O_2_ in the absence of the enzyme, also had a similar extent of degradation, indicating that a direct uncatalyzed reaction with H_2_O_2_ was primarily responsible for the degradation reaction and not the enzyme catalyzed reaction (S7-S9 Figs). In contrast to protochelin, both the ferric complex and apo forms of DFOB and PDMA were relatively unreactive with peroxidase and H_2_O_2_ (Fig 2b). Finally, protease did not significantly (< 4%) react with any of the apo siderophores or their Fe^III^-siderophore complexes (Fig 2c).

### Identification of siderophore degradation products

**Fig 3:**
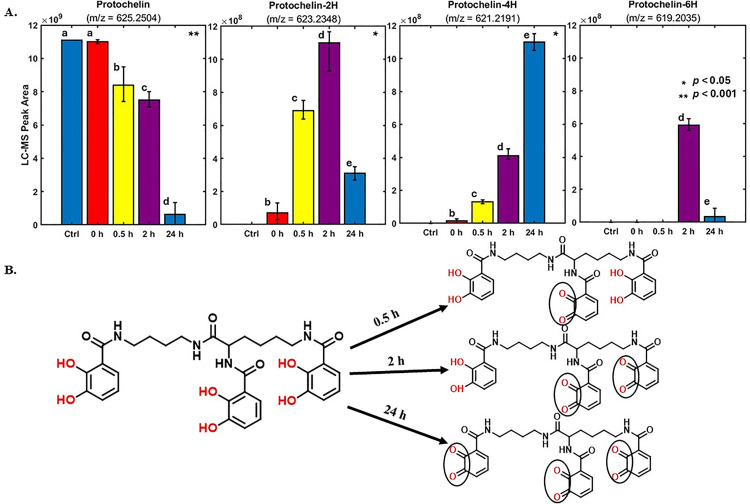
HR-LC-MS (+ ion mode) detected degradation of protochelin by phenol oxidase. (A) LC-MS peak areas for protochelin and protochelin degradation products for each of the four time points. Measurements of protochelin and protochelin degradation products were conducted simultaneously in the same samples. Error bars indicate single standard deviation from triplicate biological incubations. Statistical significance was determined using a one-way ANOVA with Tukey’s post hoc HSD test. Different letters indicate statistically significant differences between groups; asterisks (*) in the upper right corner of each plot denote the level of significance. (B) Structures of the identified protochelin degradation products, based on tandem mass-spectrometry fragmentation spectra. Fragments detected in the MS/MS spectra (see also S2 Table) are displayed. The catechol groups in protochelin and the quinone groups in the degradation products are highlighted with circles.

Sample aliquots at three time points (30 minutes, 2 h, and 24 h) were collected from triplicate reactions of phenol oxidase and peroxidase enzymes with protochelin and analyzed by LC-MS/MS. When reacted with phenol oxidase, degradation products were observed, namely the oxidation of the catechol moieties to quinones (Fig 3). The observed m/z values corresponded to the sum formulas equal to the oxidation of one or two catechol moieties (protochelin-2H, protochelin-4H) by the phenol oxidase enzyme, as previously reported [34], and LC-MS/MS analysis was in agreement with the oxidation occurring in the catechol groups (S2 Table). The oxidation of the catechol groups leads to a lessened affinity for Fe^III^ due to the loss of binding moieties. Subsequent reaction products observed were based on further reaction and degradation but accounted for only minor peak areas, including the product corresponding to the oxidation of all three catechol moieties (protochelin-6H, S2 Table). Although we were not able to obtain authentic standards for the degradation products, it is interesting to note that the sum of all peak areas of protochelin degradation products amounted to ~80% of the original protochelin standard peak area after a 30-minute reaction but only ~10% after a 24-hour reaction. Assuming the initial degradation products ionized similarly to the original protochelin structure, this suggested that these degradation products accounted for a majority of protochelin removal from the solution at 30 minutes. At longer reaction times, it is possible that smaller fragments with reduced ionization efficiency may have formed, which may account for the lower sum of the degradation product peak areas. For example, 2,3-dihydroxybenzoic acid has a more than 10-fold lower ionization efficiency than protochelin [46,49–52]. No significant differences appeared between the control and reaction samples for the protochelin m/z value at the initial sampling time (0 h), but there were significant differences at 0.5 h, 2 h, and 24 h. In contrast, the degradation products showed significant differences between the control and reaction samples across these different time points (0.5, 2 h, 24 h).

In the presence of the peroxidase enzyme and H_2_O_2_, protochelin concentrations decreased in the reaction solution (S2,S4, and S5 Figs), with most of the protochelin reacting rapidly; only ~20% of the initial protochelin concentration was detected in aliquots taken immediately after the reaction was started. However, no putative degradation products were detectable by LC-MS/MS (see discussion section for possible explanations).

### Siderophore degradation kinetics

**Fig 4:**
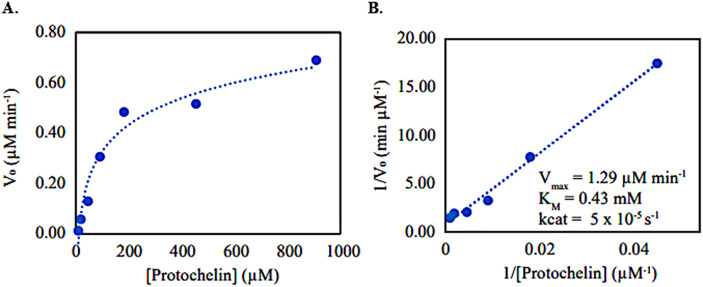
The protochelin oxidation with phenol oxidase followed (A) the Michaelis-Menten kinetics plot and (B) the Lineweaver-Burk calculation plot used to estimate the maximum rate of catalysis (Vmax) and substrate affinity (Km) for phenol oxidase activity on protochelin degradation.

The reaction between phenol oxidase and protochelin appeared to follow Michaelis-Menten kinetics (Fig 4a). The calculated K_m_ and V_max_ values from Lineweaver-Burk plots (Fig 4b) were K_m_ = 0.43 mM and V_max_ = 1.29 µM/min. Compared with other phenolics (diphenols & triphenols) reported in the literature (Table 1), our data showed the lowest K_m_ of all previously reported substrates [53]. For substrate specificity (V_max_/K_m_), previously reported V_max_/K_m_ values of phenolic substrates were greatest for small molecules, including catechol [54,55], or 4-methylcatechol [53]. Protochelin specificity was low (0.003 min^-1^), which is consistent with previously reported trends [56]. After normalizing Vmax values to enzyme activity units, the normalized Vmax value and specificity in this study are similar in magnitude to the triphenolic substrates pyrogallol (0.0071 min^-1^) [53]. On the other hand, Fe^III^-protochelin complexes did not follow Michaelis-Menten kinetics and thus were not investigated in detail.

**Table 1:** Kinetic parameters for the oxidation of protochelin substrate compared to other phenolic substrates by phenol oxidase. Normalized Vmax refers to Vmax normalized to enzyme activity units.

Substrate	Configuration	V_max_ (µM/min)	Normalized V_max_	K_m_ (mM)	V_max_/K_m_ (min^-1^)	Reference
Protochelin	tri-*o*-dihydroxy	1.29	0.0005	0.43	0.003	This study
Catechol	*o*-dihydroxy	79.5 109.9 5503 4081	0.002 0.006 0.482 0.155	5.2 8.2 4.40 3.20	0.0153 13.4 1.251 1.275	[53] [56] [54] [57]
Chlorogenic acid	*o*-dihydroxy	53.15	0.260	0.56	94.911	[58]
4-Methyl catechol	*o*-dihydroxy	125 82.1 4504 5405	0.003 0.004 0.394 0.206	6.9 18.2 6.75 1.00	0.0181 4.5 0.6673 5.41	[53] [56] [54] [57]
L-Dopa	*o*-dihydroxy	56.5 1655	0.001 0.145	4.1 4.60	0.0138 0.3598	[53] [54]
Pyrogallol	trihydroxy	8.8	0.0002	1.24	0.0071	[53]
		nd^a^		nd	nd	[56]
Caffeic acid	*o*-dihydroxy	48.2	0.0012	3.6	0.0134	[53]
Gallic acid	trihydroxy	nd^a^		nd	nd	[56]

nd^a^ = degradation was not detected.

## Discussion

### Siderophore Degradation

In this study, we examined the ability of three extracellular enzymes secreted by plants and a diverse array of microbes to degrade three representative siderophore structures. The results indicated that phenol oxidase and peroxidase were effective in degrading protochelin. In contrast, DFOB and PDMA, and their corresponding Fe^III^-siderophore complexes, showed negligible reactivity. This outcome may be explained by the absence of catechol moieties in DFOB and PDMA, which are known to react with these oxidative enzymes. Considering our reaction time and pH, where DFOB [59] and PDMA [60] are generally stable, and the enzyme concentration aligns with the practical activity range in various environments, such as the soil microbiomes [25], this result emphasizes that the degradation of siderophores by enzymes may be influenced by their structure, concentration, and binding affinities.

Further investigation into phenol oxidase activity revealed that the reduction of dissolved protochelin concentration correlated with the formation of protochelin degradation products, where the 2,3-dihydroxybenzoate binding groups were oxidized to quinone groups (Fig 3b). Our results are consistent with a previous report indicating that the oxidation of protochelin to quinones followed a reversible redox reaction mechanism that may be employed by microbes to release Fe from the Fe^III^-protochelin complexes because the quinone groups possess lesser Fe^III^ binding affinities [21]. Conversely, other studies have described the catechol groups as a “suicide substrate” for the tyrosinase enzyme, causing irreversible inactivation during catechol oxidation. This inactivation occurs through two mechanisms: (a) an attack on the nucleophilic group vicinal to the enzyme active site by quinone oxidation products [61], followed by (b) a free radical attack via reactive oxygen species formed during oxidation on the enzyme’s active site [62,63]. However, no evidence of this phenomenon was observed in our study, as the enzyme did not precipitate out of the solution.

The kinetic values of V_max_ = 1.29 µM min^-1^ and substrate specificity V_max_/K_m_ = 0.003 min^-1,^ as well as normalized V_max_ values from our results, compared with other phenols (including diphenols and triphenols) reported in the literature, suggested that protochelin degrades slowly and with unspecific affinity compared to smaller phenolic compounds. Earlier reports on phenol oxidases propounded that the substrate-binding site of phenol oxidases has a high affinity for smaller o-diphenols [56] such as catechol [57,64,65], 4-methyl catechol [66], or L-dopa [55,67], and less affinity for the larger o-diphenols [53] and triphenols [53,56]. Based on this trend, a plausible explanation for our results could be steric hindrance from protochelin’s triscatecholamide structure and long-tail aliphatic amides.

A diversity of phenol oxidases are known to occur widely in nature, produced by fungi (*Agaricus bisporus* [68], *Basidiomycota* [69]), bacteria (*Azotobacter chroococcum* [25,70]), and plants (apricot [71], Chinese Toon [72], mamey [73], Hemşin apple [74], and lentil sprouts [75]). It is possible that these phenol oxidase enzymes may have a range of reactivities with protochelin depending on the enzyme sources in the environment. Although we did not study protochelin siderophore with other types of phenol oxidases (catechol oxidases, aurone synthases) or in the presence of other phenol substrates, our results showed that such enzyme-siderophore reactions may occur and thus may impact environmental Fe complexation and transport.

Additionally, Fe^III^-protochelin complexes degraded less (~50%) when reacted with phenol oxidase, suggesting that Fe^III^ binding may have restricted direct interaction of the catechol groups with enzyme active sites, thereby reducing the rate of reaction for protochelin degradation (S6 Fig). A prior review on microbial siderophore-mediated transport noted that Fe^III^ binding by the triscatecholate siderophore enterobactin enhanced stability and improved resistance to potential degrading enzymes [3]. Because protochelin has a high affinity for Fe^III^ and can bind over a wide pH range [21,48], we can assume minimal dissociation of complexes, resulting in potential protection of complexes from enzymic degradation under environmentally common pH values.

The peroxidase enzyme and co-substrate H_2_O_2_ removed protochelin from the solution rapidly (S4 and S5 Figs). This outcome was not surprising, as peroxidases have previously been reportedly utilized in the biodegradation of phenolic contaminants in bioremediation studies [69,76]. However, we did not observe any degradation products by LC-MS or LC-UV-vis, and attempts to recover protochelin or its degradation products by solid phase extraction with methanol were unsuccessful (not shown). This absence of degradation products was not likely caused by protochelin residues being trapped in the enzyme’s binding sites, as no precipitates were observed in our experiments. Furthermore, the protochelin peak also rapidly diminished when reacted with H_2_O_2_ in the absence of peroxidase (S7 Fig). In contrast, degradation was slow when only peroxidase was present in the absence of H_2_O_2_ (S8 Fig). Thus, our results indicate that H_2_O_2_ promotes degradation even without catalysis by peroxidase. Because H_2_O_2_ may be present in (sub)-micromolar concentrations in soil [77] and aquatic environments [78,79], our observations may have highlighted a potential role of H_2_O_2_ in the degradation of the catechol-type siderophores.

In contrast to protochelin, the reaction of peroxidase (and H_2_O_2_) with DFOB and PDMA was insignificant. This outcome may be anticipated due to the lack of catechol groups in these siderophores and, consequently, a much higher stability towards oxidation by H_2_O_2_. Moreover, the hydroxamate groups in DFOB have been noted previously as inhibitors of peroxidases [80–82], where the hydroxamate moieties may form hydrogen-bond interactions with the backbone of enzymes, which is critical for their selectivity as enzyme inhibitors [83].

Protease showed no significant activity with any of our siderophores, despite being reported to play a crucial role in catalyzing the initial step of protein degradation in cellular nitrogen metabolism, for example, in the rhizosphere [84,85]. A plausible explanation for the low reactivity of protease with protochelin, DFOB, and PDMA may lie in the modified structures of the peptide monomers, which make up these siderophores, and which render them poor substrates for protease activity. In our experiments, an increase in protease concentrations established that the observed lack of reactivity is likely due to enzyme specificity and not insufficient enzyme concentration. Additionally, the hydroxamate groups present in DFOB are recognized as effective inhibitors of proteases [86,87]. Moreover, it was reported that the inhibition of protease activity was notably increased with higher DFOB production by *Streptomyces pilosus* ATCC 19797 in soybean medium [88], consequently enhancing their carbon source. Intracellular protease activity is crucial for processes like protein and siderophore degradation [38,89], signaling [90], and cellular turnover [91], whereas extracellular proteases are mostly involved in plant immunity [92] and interactions with the environment [93,94]. This process specifically, may in part explain why our extracellular protease showed nearly inert behavior toward substrates containing catechol, hydroxamate, and carboxylate groups.

The proline analog of deoxymugineic acid (PDMA) demonstrated minimal reactivity with the three extracellular enzymes (~1–4% degradation). PDMA is a synthetic phytosiderophore analog and does not represent bacterial or fungal carboxylate siderophore structures, but it is an analog of phytosiderophores, such as deoxymugineic acid [95,96]. PDMA demonstrates greater stability than its natural counterpart, deoxymugineic acid (DMA), due to structural modifications [97]. This enhanced stability is in part why PDMA was chosen, and because of recent interest in its use as a new effective Fe fertilizer [60,98–100]. In particular, its relative stability may be attributed to the substitution of the traditional azetidine-2-carboxylic acid group in deoxymugineic acid (DMA) with an L-Proline analog [40,41], which helps decrease its degradation rate by microorganisms. Our results with extracellular enzymes are consistent with this increased structural stability. Additional studies with other compounds in the mugineic acid family may better constrain the range of susceptibilities of phytosiderophores to degradation by extracellular enzymes.

### Impact of siderophore degradation on iron cycling

The environmental distribution and activities of extracellular enzymes are influenced by several factors, including water table depth [101], organic carbon content (SOC) [68], pH [102], and soil depth [103]. Importantly, most enzymes are inducible, meaning that plants and microbes produce these extracellular enzymes in response to the availability of substrate and/or product. Therefore, this makes enzyme activities dynamic, changing with the concentrations of substrates or products in various microbiomes. Soil, for example, can show relatively high activities of phenol oxidase [104], peroxidase [105], and protease [106] due to the presence of abundant inducible substrates, such as proteins/peptides [107,108], and lignin, along with other phenolic compounds [25]. Similarly, as Fe availability decreases, plants and microbes experience abiotic stress. Consequently, this stress stimulates the exudation of siderophores, which are typically found at submicromolar concentrations extracted from soil [51], and could interact with these oxidative extracellular enzymes, leading to degradation. However, the presence of other compounds, such as phenolics, could potentially interfere with the degradation process, for example, by competing for enzyme active sites, altering redox reactions, or scavenging reactive radicals, especially considering the low substrate specificity of the siderophores we tested. Given their importance in organic matter decomposition and nutrient cycling, these extracellular enzyme activities related to siderophore degradation may likely interact with the siderophores released by plants or microbes in response to Fe deficiency, thereby affecting competition for Fe by microbiomes. Further studies are required to better establish the impact of phenol oxidases and peroxidases on catechol siderophore degradation in various microbiomes, such as root environments or natural water systems.

## Supporting information

S1 Appendix Enzyme activity assays. (PDF)

S1 Table Siderophore standards, ionization mode, and *m/z* values used in LC-MS analysis with Single-Ion-Monitoring. (PDF)

S2 Table MS/MS fragmentation of protochelin and protochelin reaction products referenced in Fig 3. (PDF)

S3 Table Protochelin degradation at different time points of the 24-hour reaction with phenol oxidase. **Characteristic MS/MS fragments are shown in** **S2 Table**.
(PDF)

S1 Fig Reduction of protochelin parent peak after reaction with phenol oxidase for 30 minutes, 1,2, and 24 hours from LC-MS analysis. (TIF)

S2 Fig Reduction of protochelin parent peak after reaction with peroxidase immediately after starting the reaction (0 h), 30 minutes (0.5 h), and 24 hours from LC-MS analysis. (TIF)

S3 Fig Plot of the change in absorbance spectrum of protochelin in solution over a 24 h period as ligand degradation occurs after reaction with phenol oxidase enzyme. Conditions: pH = 7.0, c = 100 µM protochelin siderophore, T = 25˚C.
(TIF)

S4 Fig Spectrophotometric changes in the absorbance of protochelin in the solution over (A) 60 seconds and (B) 60 minutes, as ligand degradation occurs after reaction with peroxidase enzyme and H_2_O_2_ as a cosubstrate. Conditions: pH = 6.79, c = 100 µM protochelin siderophore, T = 25˚C.
(TIF)

S5 Fig Plot of the change in absorbance spectrum of protochelin in solution over a 24 h period as ligand degradation occurs after reaction with peroxidase enzyme and H_2_O_2_ as a cosubstrate. Conditions: pH = 6.88, c = 100 µM protochelin siderophore, T = 25˚C.
(TIF)

S6 Fig Schematic showing (A) phenol oxidase–Protochelin interaction without Fe, indicating easier binding to enzyme active sites, whereas (B) Fe inhibits direct interaction of phenol oxidase and chelating groups of Protochelin-Fe chelate. (TIF)

S7 Fig Plot of the change in absorbance spectrum of protochelin in solution over a 24 h period as ligand degradation occurs after reaction with only H_2_O_2_. Conditions: pH = 7.12, c = 100 µM protochelin siderophore, T = 25˚C.
(TIF)

S8 Fig Spectrophotometric changes in the absorbance of protochelin in the solution over a 24 h period, as slow ligand degradation occurred after reaction with just the peroxidase enzyme. Conditions: pH = 6.82, c = 100 µM protochelin siderophore, T = 25˚C.
(TIF)

S9 Fig Spectrophotometric changes in the absorbance of protochelin in the solution over a 24 h period, as rapid ligand degradation occurred after reaction with just the H_2_O_2_ (Control 1), with heat-inactive peroxidase enzyme and H2O2 (Control 3), whereas a slower absorbance reduction was observed with only peroxidase enzyme (Control 4). Conditions: pH = 6.81, c = 100 µM protochelin siderophore, T = 25˚C.
(TIF)
