# Supplementary material for: Degradation of catecholate, hydroxamate, and carboxylate model siderophores by extracellular enzymes
Source: PLoS One. 2025 Aug 19;20(8):e0330432. doi: 10.1371/journal.pone.0330432 (PMC12364333; doi:10.1371/journal.pone.0330432)
Supplement: S1 Appendix — (PDF) [file pone.0330432.s001.pdf]

S1 Appendix. Enzyme activity assays.

(a) Phenol oxidase: Three milliliters of the reaction mixture to determine the phenol oxidase activity consisted of 1 mL 50 mM potassium phosphate buffer (pH 7.0), 1 mL 0.001 M L-tyrosine, and 900 µL of ultra-pure water. The enzyme assay sample reaction in a 15 mL centrifuge tube was left to stand for 4-5 minutes to be oxygenated before adding 100 µL of 200-400 units/mL of phenol oxidase enzyme. The absorbance at 280 nm was monitored for 10 minutes using a Thermo-scientific Evolution 201 UV-Vis spectrophotometer (Waltham, MA, USA). Enzyme activity was calculated based on the rate of absorbance increase, proportional to enzyme concentration, and linear during 5-10 minutes after an initial lag. One unit causes a change in absorbance at 280 nm of 0.001 per minute at 25°C, pH 6.5-7.4, under the specified conditions.

(b) Peroxidase: Three milliliters of the reaction mixture to determine the peroxidase activity consisted of 320 µL 100 mM potassium phosphate buffer, pH 7.01, 160 µL 0.50% hydrogen peroxide, 320 µL 0.1 M pyrogallol, 2.1 mL of ultra-pure water, and 100 µL peroxidase enzyme solution. The blank sample consisted of 320 µL 100 mM potassium phosphate buffer, pH 7.01, 160 µL 0.50% hydrogen peroxide, 320 µL 0.1 M pyrogallol, and 2.2 mL ultrapure water only. In the 3.00 mL reaction mix, the final concentrations are 14 mM phosphate buffer (pH 7.01), 0.16 M 0.027% (v/v) hydrogen peroxide, 0.1 M 0.5% (w/v) pyrogallol, and 0.45–0.75-unit peroxidase enzyme. The absorbance at 420 nm was monitored for 3 minutes using a UV-Vis spectrophotometer, as described above. The enzymatic activity was calculated by considering the linear part of the curve (optical density vs. time). One unit of enzyme activity was defined as the amount of enzyme that produces an absorbance increase of 0.001/minute/mL at 25ºC. This unit is equivalent to ~18 µM units per minute at 25 °C. The enzymatic activity was calculated as the mean of three experiments for all tested conditions.

(c) Protease: The protease activity was determined by a method previously used by Mayerhofer et al. (1973). The final concentration for the assay mixture: 1 mL 8.6 mM borate solution, 1 mL (5.1 mg/mL) (0.6 %) casein solution, 500 µL 0.29 mM calcium acetate solution, and 500 µL 30 Units/mL protease enzyme solution. The reaction mixture was filtered after 10 minutes, and the optical density of the filtrate at 275 nm (OD test) was measured using UV-Vis spectrophotometry, as described above. The protease activity was calculated in activity units (U). One activity unit was defined as the amount of enzyme that is required to hydrolyze casein to give 1 µM of tyrosine in 1 min at pH 7.0 and a temperature of 25 °C. One unit causes an increase of optical density at 275 nm corresponding to one micromole of tyrosine per minute.
