# Supplementary material for: Degradation of catecholate, hydroxamate, and carboxylate model siderophores by extracellular enzymes
Source: PLoS One. 2025 Aug 19;20(8):e0330432. doi: 10.1371/journal.pone.0330432 (PMC12364333; doi:10.1371/journal.pone.0330432)
Supplement: S1 Table — (PDF) [file pone.0330432.s002.pdf]

S1 Table. Siderophore standards, ionization mode, and *m/z* values used in LC-MS analysis with Single-Ion-Monitoring (see methods).

**Siderophore**	**Ionization**	**Exact mass (M)**	***m/z* (MH$^+$)**	***m/z* (M-H)$^-$**	***z***
DFOB	Positive	560.3534	561.4		1
DFOB – O	Positive	544.3585	545.4		1
DFOB – 2O	Positive	528.3635	529.4		1
FeDFOB	Positive	613.2648	614.3		1
PDMA	Positive	318.1427	319.1		1
FePDMA	Positive	371.0542	372.1		1
Protochelin	Negative	624.2431		623.2	1
Protochelin – 2H	Negative	622.2256		621.2	1
Protochelin – 4H	Negative	620.2191		619.2	1
FeProtochelin	Negative	677.1546		676.2	1
