# Supplementary material for: Degradation of catecholate, hydroxamate, and carboxylate model siderophores by extracellular enzymes
Source: PLoS One. 2025 Aug 19;20(8):e0330432. doi: 10.1371/journal.pone.0330432 (PMC12364333; doi:10.1371/journal.pone.0330432)
Supplement: S2 Table — (PDF) [file pone.0330432.s003.pdf]

S2 Table. MS/MS fragmentation of protochelin and protochelin reaction products referenced in Fig 3.

**Protochelin**	
Precursor *m/z*	625.2504
Fragment *m/z*	Intensity (%)
489.2341	1.3
353.2181	7.1
265.1185	3.7
237.2022	0.8
225.1232	53.5
208.0966	31.1
137.0231	9.3
84.0807	100.0

**Protochelin-2H**	
Precursor *m/z*	623.2348
Fragment *m/z*	Intensity (%)
623.2348	3.1
605.2230	2.4
469.2078	37.4
442.1973	57.9
354.0965	28.5
341.1137	25.9
217.2021	17.5
129.1023	11.3
89.1072	44.7
84.0807	100.0
72.0807	63.6

**Protochelin-4H**	
Precursor *m/z*	621.2191
Fragment *m/z*	Intensity (%)
621.2191	23.4
533.1181	2.3
487.2117	12.1
468.1761	7.2
485.2029	100.0
440.1808	48.1
371.1128	8.3
369.1069	47.7
343.3286	25.3
260.0550	17.1
155.1542	13.2
89.1072	18.6
84.0807	93.2
72.0807	76.4

**Protochelin-6H**	
Precursor *m/z*	619.2035
Fragment *m/z*	Intensity (%)
619.2035	4.8
591.2079	37.3
502.1228	4.6
440.1794	31.4
439.1732	100.0
438.1652	32.1
373.1264	45.4
372.1186	23.2
218.0809	18.3
137.1197	7.6
89.1072	41.3
84.0807	62.1
72.0807	38.4
