# Supplementary material for: Degradation of catecholate, hydroxamate, and carboxylate model siderophores by extracellular enzymes
Source: PLoS One. 2025 Aug 19;20(8):e0330432. doi: 10.1371/journal.pone.0330432 (PMC12364333; doi:10.1371/journal.pone.0330432)
Supplement: S3 Table — Characteristic MS/MS fragments are shown in S2 Table. (PDF) [file pone.0330432.s004.pdf]

S3 Table. Protochelin degradation at different time points of the 24-hour reaction with phenol oxidase. Characteristic MS/MS fragments are shown in S2 Table.

m/z	***Sampling time point (h)***	**RT (min)**	**Peak Area**	**Sum formula**
625.2504	0	4.9	1.10E+10	$C_{31}H_{36}N_4O_{10}$
	0.5	4.9	8.40E+09	
	2	4.9	7.50E+09	
	24	4.9	6.20E+08	
623.2348	0	4.4	7.00E+07	$C_{31}H_{34}N_4O_{10}$
	0.5	4.4	6.90E+08	
	2	4.4	1.10E+09	
	24	4.4	3.10E+08	
621.2191	0	4.3	1.60E+07	$C_{31}H_{32}N_4O_{10}$
	0.5	4.4	1.30E+08	
	2	4.3	4.10E+08	
	24	4.3	1.10E+09	
