# Supplementary figures and images for: Degradation of catecholate, hydroxamate, and carboxylate model siderophores by extracellular enzymes
Source: PLoS One. 2025 Aug 19;20(8):e0330432. doi: 10.1371/journal.pone.0330432 (PMC12364333; doi:10.1371/journal.pone.0330432)

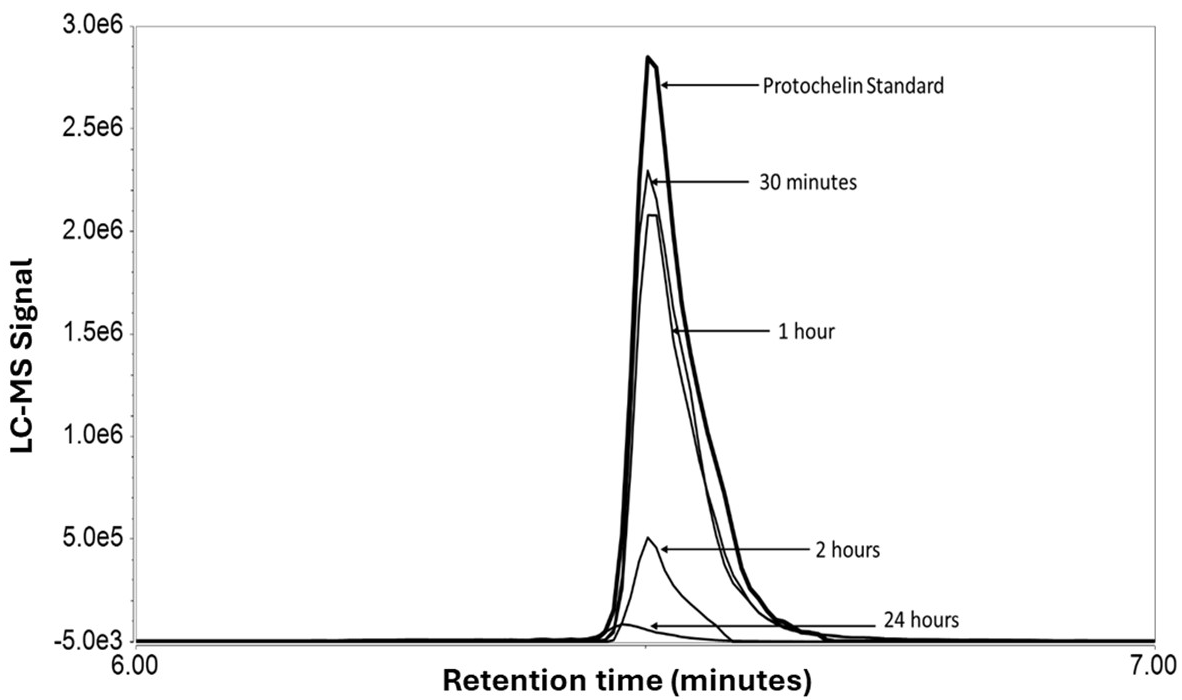

Supplement: S1 Fig — (TIF) [file pone.0330432.s005.tif]

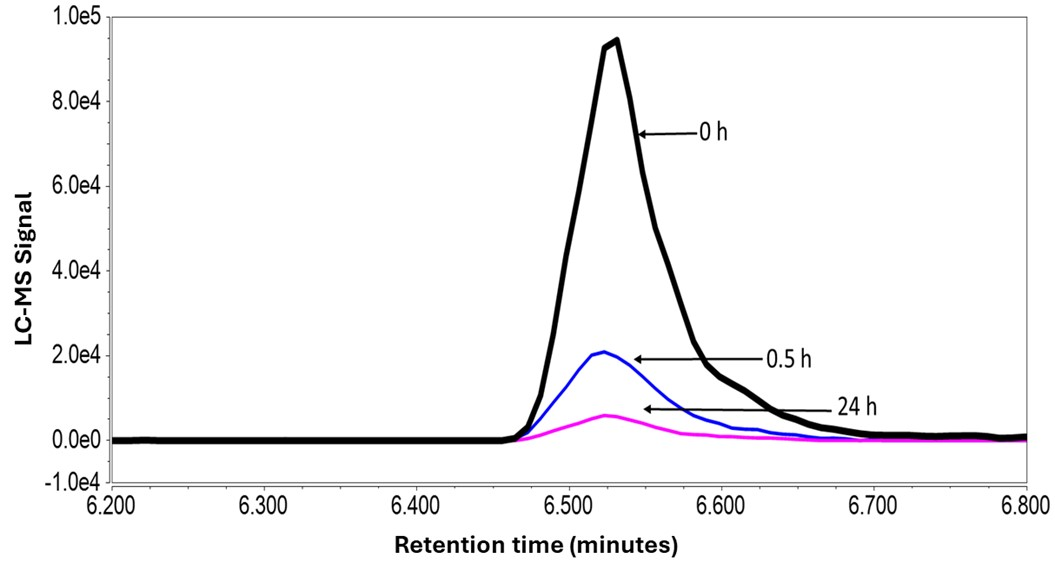

Supplement: S2 Fig — (TIF) [file pone.0330432.s006.tif]

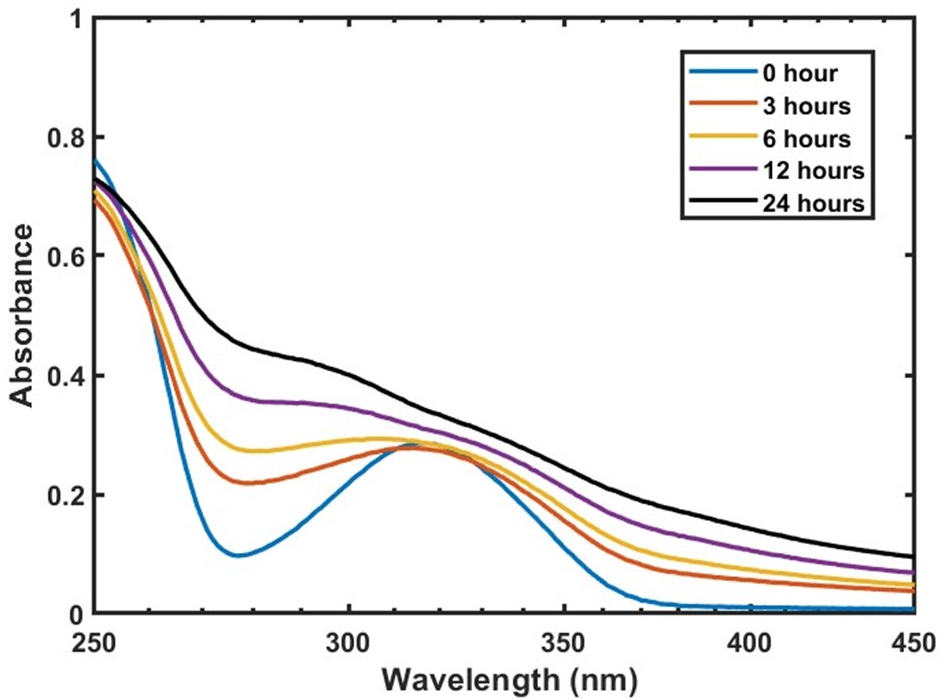

Supplement: S3 Fig — Conditions: pH = 7.0, c = 100 µM protochelin siderophore, T = 25˚C. (TIF) [file pone.0330432.s007.tif]

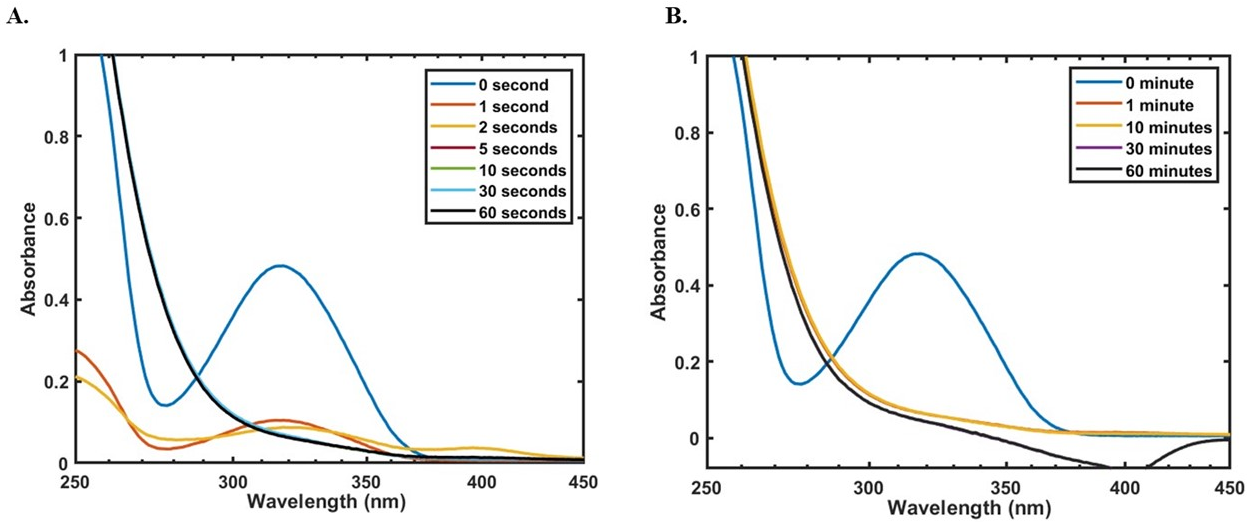

Supplement: S4 Fig — Conditions: pH = 6.79, c = 100 µM protochelin siderophore, T = 25˚C. (TIF) [file pone.0330432.s008.tif]

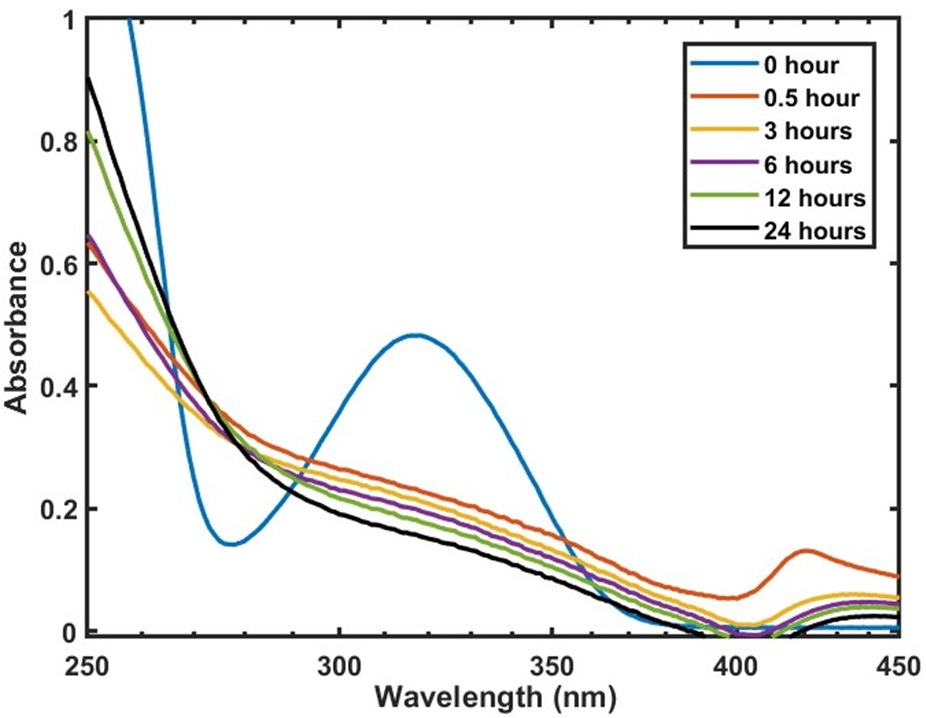

Supplement: S5 Fig — Conditions: pH = 6.88, c = 100 µM protochelin siderophore, T = 25˚C. (TIF) [file pone.0330432.s009.tif]

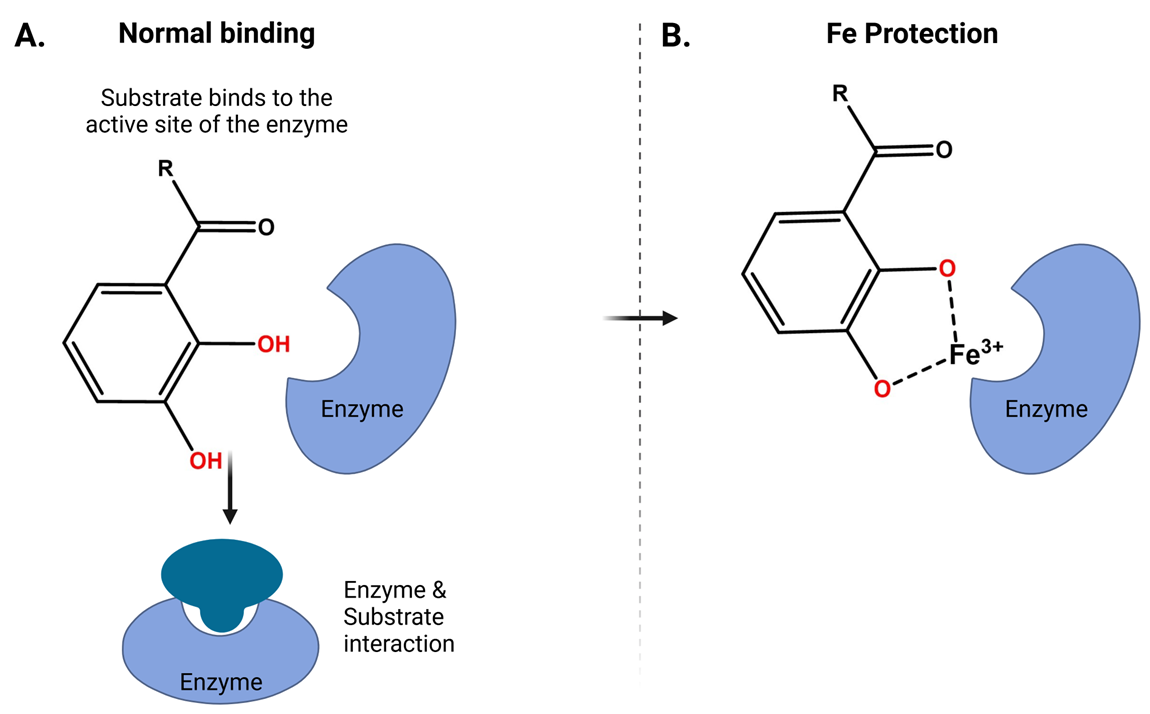

Supplement: S6 Fig — (TIF) [file pone.0330432.s010.tif]

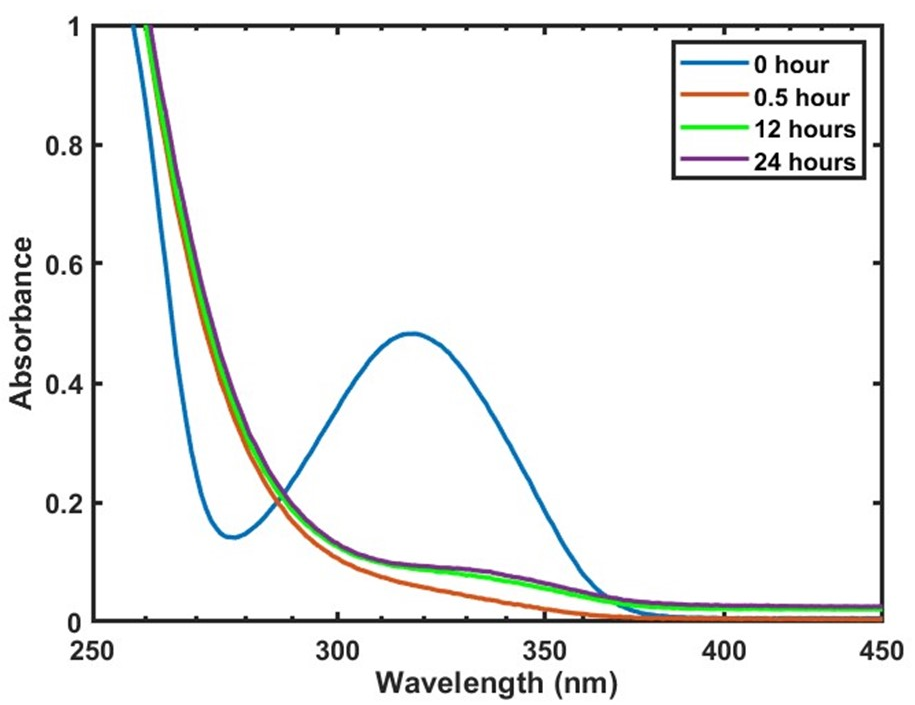

Supplement: S7 Fig — Conditions: pH = 7.12, c = 100 µM protochelin siderophore, T = 25˚C. (TIF) [file pone.0330432.s011.tif]

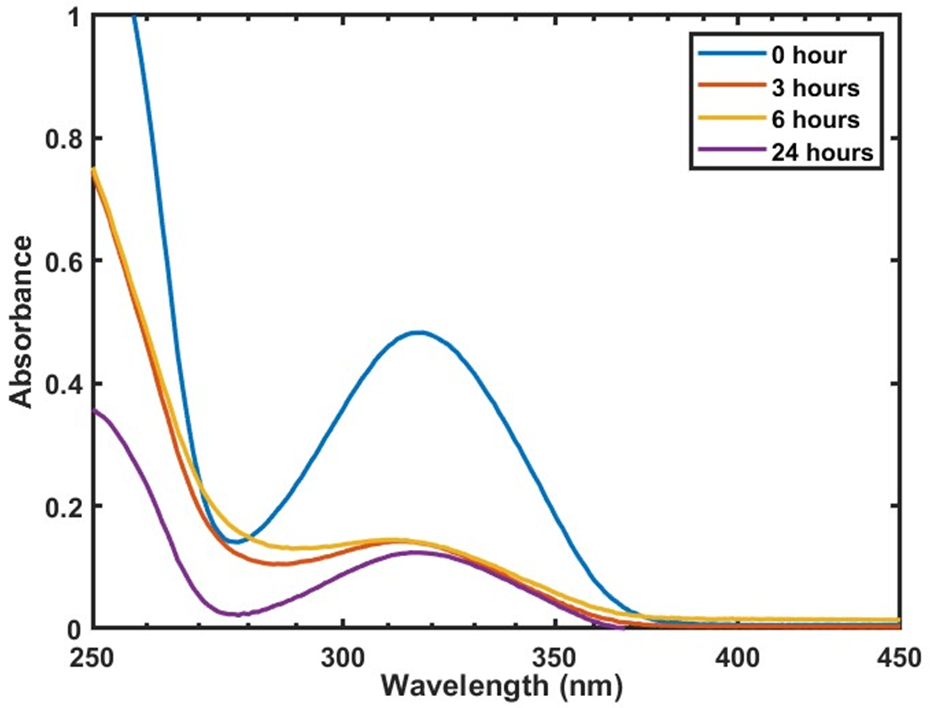

Supplement: S8 Fig — Conditions: pH = 6.82, c = 100 µM protochelin siderophore, T = 25˚C. (TIF) [file pone.0330432.s012.tif]

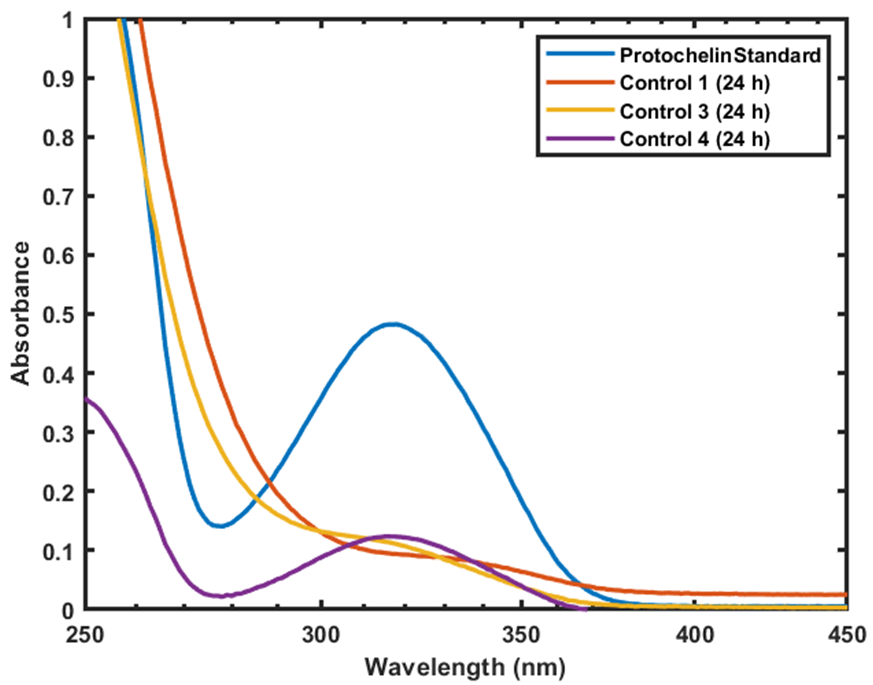

Supplement: S9 Fig — Conditions: pH = 6.81, c = 100 µM protochelin siderophore, T = 25˚C. (TIF) [file pone.0330432.s013.tif]
